# Relapse rates after elective discontinuation of anti-TNF therapy in rheumatoid arthritis: a meta-analysis and review of literature

**DOI:** 10.1186/s41927-019-0058-7

**Published:** 2019-03-08

**Authors:** Arduino A. Mangoni, Fahdah Al Okaily, Hani Almoallim, Seham Al Rashidi, Reem Hamdy A. Mohammed, Amal Barbary

**Affiliations:** 1 Department of Clinical Pharmacology, Flinders University and Flinders Medical Centre, Bedford Park, SA 5042 Australia; 2 Prince Sultan Military Medical City, Riyadh, Saudi Arabia; 3 Department of Medicine, Umm Alqura University, Jeddah, Kingdom of Saudi Arabia; 4 Rheumatology and Clinical Immunology, Department of Rheumatology and Rehabilitation, School of Medicine, Cairo University Hospitals, Cairo, Egypt; 5 Internal Medicine Department, Alhada Armed Forces Hospital, Taif, Kingdom of Saudi Arabia; 6 Department of Rheumatology and Rehabilitation, Tanta University Faculty of Medicine, Elgesh Street, Tanta, Gharbeia Egypt

**Keywords:** DMARDs (synthetic), TNF-α inhibitors, Rheumatoid arthritis, Treatment discontinuation, Relapse

## Abstract

**Background:**

Inhibitors of tumor necrosis factor alpha (TNF-α) are current mainstay of therapies for rheumatoid arthritis (RA). The decision when to withdraw TNF-α inhibitors after achieving remission and the incidence of relapse rates with elective discontinuation are both important questions that demand intense survey in these patients. In this meta-analysis we aimed to estimate the magnitude of relapse rate after elective TNF-α inhibitor discontinuation in RA patients with remission.

**Methods:**

Systematic searches of PubMed/MEDLINE, Cochrane Library databases, grey literature (unpublished and ongoing trials) from the WHO International Clinical Trials Registry Platform and the US National Institutes of Health were performed for studies reporting the outcomes of elective discontinuation of TNF-α inhibitor in RA patients after remission. Random-effects models for meta-analyses were conducted on extracted data.

**Results:**

Out of 390 references screened, 16 RCTs were included. Meta-analysis of 1264 patient data revealed a relapse rate of 0.47 (95% CI 0.41–0.54). Sensitivity analysis showed that none of the studies had higher influence on the results.

**Conclusions:**

Almost half of all the RA patients in remission relapse after elective TNF-α inhibitor discontinuation. This information might be useful when considering this management option with individual patients.

## Background

Rheumatoid arthritis (RA) is a chronic multisystem autoimmune inflammatory disease that leads to significant joint inflammation with damage and deformity. The disease has an annual incidence of three cases per 10,000, and a prevalence of 1%, increasing with age and peaking between the ages of 35 and 50 years [1]. RA affects all populations, with few ethnic variations (e.g., 5–6% in some Native American groups vs. 0.8% in black-Caribbeans) [1, 2]. Women are affected three times more often than men however sex differences tend to diminish in older age groups with a female to male ratio of 2:1 after the fifth decade of life [1, 3].

The pharmacological therapies for RA comprise nonsteroidal anti-inflammatory drugs (NSAIDs), corticosteroids and the disease-modifying antirheumatic drugs (DMARDs). The DMARDs include non-biologic (e.g. methotrexate) and biologic agents (TNF-α inhibitor drugs and non TNF-α biologics) that halt the progression of RA by reducing inflammation, preventing joint damage and maintaining the integrity of joints [1].

The TNF-α inhibitors etanercept, infliximab, certolizumab pegol, adalimumab, and golimumab, are a class of biologic DMARDs directed towards the TNF-α proinflammatory cytokine, and can be administered either subcutaneously or intravenously. TNF-α inhibitors have an established role in the induction and maintenance of remission in patients with RA [4]. However, suppression of TNF-α also leads to a range of adverse effects including the emergence of antinuclear antibodies (ANAs), generation of antibodies against these compounds, infections (including tuberculosis), increased risk of cancer, heart failure, demyelinating disorders, and bone marrow suppression [5]. Immunogenicity has been shown to occur in patients receiving adalimumab and infliximab, potentially leading to decreased drug efficacy [6]. The risk of developing such adverse events, the inconvenience of parenteral administration, and the high cost of these agents raised the possibility of elective withdrawal in RA patients with a considerable disease-free period and in whom the treatment objectives were achieved. However, the continuation of TNF-α inhibitor therapy, in RA patients in remission or low disease activity, increased the probability of sustained response (whether remission or low disease activity) and retarded radiographic progression in a number of published meta-analyses [7, 8]. Furthermore, the incidence of serious adverse events, serious infection, malignancy, and scores of improvement of tender and swollen joints were not significantly different between strategies favoring continuation and those with elective discontinuation after remission with almost half of the patients withdrawing biologicals maintaining low disease activity [6, 7].

Though elective TNF-α inhibitor discontinuation is justified in several RA patients, there is a lack of sufficient data to guide the decision. Further, the course of action post-withdrawal also remains to be understood, although a decision based on sustained remission has been proposed [9]. Nonetheless, a consensus about patient selection and the timing of withdrawal remains to be reached.

As an initial step to address these issues, we investigated whether TNF-α inhibitors can be withdrawn in general. To this end, we performed a meta-analysis of studies investigating the relapse rates after elective withdrawal of TNF-α inhibitor therapy in RA patients.

## Methods

### Inclusion criteria

1- Studies that included RA patients classified according to either the American Rheumatism Association 1987 revised criteria for the classification of RA or the 2010 American College of Rheumatology (ACR)/European League Against Rheumatism (EULAR) RA classification criteria were used [10, 11].

2- Studies that investigated the relapse rate following elective withdrawal of TNF-α inhibitors (adalimumab, certolizumab pegol, etanercept, golimumab, and infliximab) as a first line or non first line biologic in patients with RA.

### Literature search

Literature search from the earliest available date to March 2016 was performed in PubMed/MEDLINE and the Cochrane Library databases, and grey literature (unpublished and ongoing trials) was assessed from the WHO International Clinical Trials Registry Platform (http://www.who.int/ictrp/en/) and the US National Institutes of Health (https://clinicaltrials.gov/) using the keywords “adalimumab” or “infliximab” or “golimumab” or “certolizumab pegol” or “etanercept” or “biological Products” and “Arthritis, Rheumatoid” and “withdrawal” or “withdrawn” or “discontinue” or “discontinuation” or “stop” or “stopped.” The possibilities of finding all relevant publications were increased by not setting the limitations on language, year, or status during the initial search. The reference lists of included articles were also screened manually for additional studies. The commentaries and conference proceedings, however, were excluded.

### Data extraction and methodological quality assessment

The reviewers (F. Alokaily & S. AlRashidi) independently screened for potentially relevant article titles and abstracts based on the inclusion criteria. Also, full text articles were retrieved wherever necessary. Authors were involved independently in all stages of study selection and data extraction.

The methodological quality of each selected randomized study was assessed by the modified Jadad scale system [12]. The criteria for evaluation were: randomization, blinding, withdrawals, dropouts, inclusion/exclusion criteria, adverse effects, and statistical analysis. The evaluated scores of studies ranged from 0 to 5 points. A study with a score of ≥3 was considered as of good quality. The quality of non-randomized trials was evaluated by CASP (The Critical Appraisal Skills Programme) checklist for Cohort study [13]. If ≥5 of the questions in CASP provided positive results about a non-randomized trial, then the study was considered high quality.

### Statistical analysis

Meta-analysis of the included studies was conducted using relapse rate with 95% confidence interval (CI). Pooled relapse rate with 95% confidence interval was estimated incorporating fixed-effects model (based on the Mantel-Haenszel method) or random-effects model (based on the DerSimonian-Laird method) [14, 15]. Presence of heterogeneity was tested by *Q*-statistic [16] and quantified by *I*^*2*^-index [17]. *Q*-statistic evaluated the presence of heterogeneity among the selected studies. Significant heterogeneity was marked by *p-value* of less than 0.05. The *I*^*2*^-index quantified the amount of heterogeneity among the selected studies. *I*^*2*^values of 25, 50 and 75% suggested low, moderate and high degrees of heterogeneity, respectively. If there was no significant heterogeneity fixed-effects model was used; otherwise, random-effects model was used.

Sensitivity Analysis: To investigate the validity and robustness of meta-analysis the leave-one-out sensitivity method was applied to establish the robustness of the meta-analysis results.

Cumulative meta-analysis: Studies were included chronologically to identify the consistency in the result of selected studies.

Publication Bias: Publication bias was examined visually by producing a funnel plot where the standard error of the estimated event rates was plotted against the logit event rates [18]. If asymmetry in funnel plot was observed the trim and fill method was adopted to assess the impact of publication bias [19]. Ideally, approximately similar number of studies are expected to fall on either side of the plot. In case of asymmetry in the number of studies plotted, the trim and fill method plots the possibly missing studies. The number of missing studies plotted correlates proportionately with the publication bias.

All meta-analyses were executed using the Comprehensive Meta-analysis Software, Version 2 (Biostat, Englewood, NJ, USA).

## Results

**Fig. 1:**
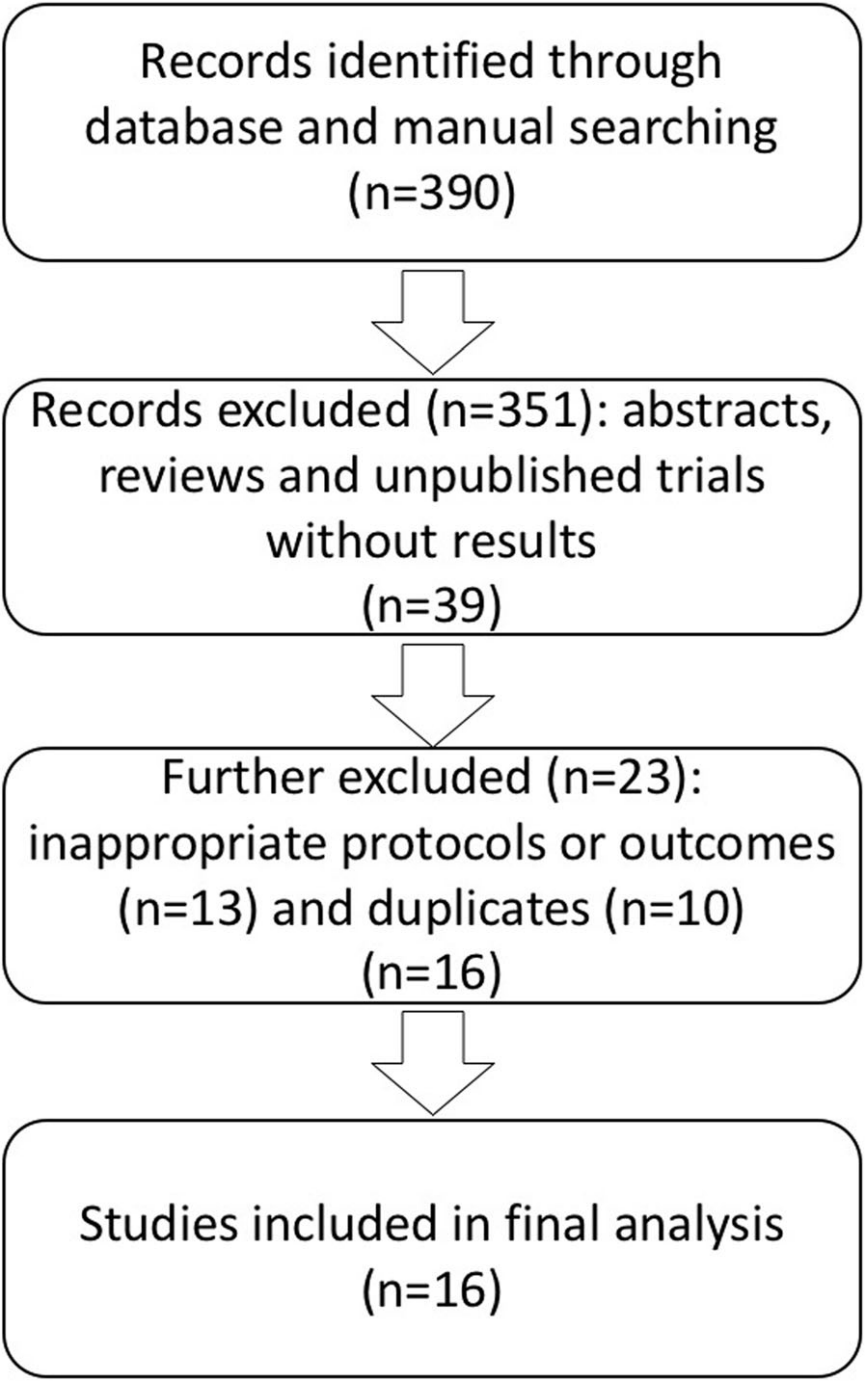
Flowchart for identification of studies used

A total of 390 citations were identified from all databases. Screening excluded 351 abstracts, reviews and unpublished trials without results. After a further screening of titles and abstracts, 13 citations were excluded because of inappropriate protocols or outcomes. Out of 26 studies, 10 appeared to be duplicates. Thus, 16 studies (*n* = 13, published and *n* = 3, unpublished) were finally eligible (Fig. 1; Tables 1 and 2). Ten studies were conducted in Europe, five were conducted in Japan, and one was conducted in Europe, Latin America, Asia and Australia. Seven studies were randomized, seven were observational/prospective, whereas the remaining two were retrospective (Table 1). Criteria for TNF-α inhibitor withdrawal were based on the DAS28/44 scoring system in 11 studies, clinical parameters in one study, and undefined in four studies. Duration of follow-up after withdrawal was ≥12 months in 12 studies, and < 12 months in the remaining four studies (Table 2).

**Table 1:** Study characteristics

	Study characteristics			
S.No	Study	Region	Type of study	Source of funding
1	Quinn et al., 2005	Europe (United Kingdom)	Randomized controlled trial	Not disclosed
2	Nawata et al., 2008	Asia (Japan)	Observational/prospective	Government
3	Brocq et al., 2009	Europe (France)	Observational/prospective	Not disclosed
4	Tanaka et al., 2010	Asia (Japan)	Observational/prospective	Government
5	van den Broek et al., 2011	Europe (Netherland)	Randomized, multicenter, single blind	Not disclosed
6	van der Maas et al., 2012	Europe (Netherland)	Observational/prospective	Not disclosed
7	Harigai et al., 2012	Asia (Japan)	Observational/retrospective	Government
8	Hirata et al., 2013	Asia (Japan)	Observational/prospective	Government
9	Smolen et al., 2013	Europe, Latin America, Asia, Australia	Randomized controlled trial	Industry
10	Iwamoto et al., 2014	Asia (Japan)	Observational/prospective	Not disclosed
11	Kádár et al., 2014	Europe (Hungary)	Retrospective, multicenter, cohort	Government
12	Tanaka et al., 2015	Europe (Hungary)	Observational/prospective	Government
13	Moghadam et al., 2016	Europe (Netherland)	Randomized controlled trial, multicenter, open label	Government
14	NCT00808509 (ADMIRE)	Europe (Sweden)	Randomized, parallel group, open label	Industry
15	NCT00858780 (DOSERA)	Europe (Denmark, Finland, Hungary, Iceland, Norway, Sweden)	Randomized controlled trial, double blind	Industry
16	NCT00858780 (DOSERA) b	Europe (Denmark, Finland)	Randomized controlled trial, double blind	Industry

**Table 2:** Patient, intervention and outcome characteristics of the studies

		Patient characteristics			Intervention characteristics							
*S.No*	*Study*	*Criteria for TNFi withdrawal*	*Duration of RA during TNFi withdrawal, years*	*DAS28 during withdrawal*	*TNFi*	*DMARDs*	*Duration of dosage, months*	*Post-withdrawal follow-up time, months*	*DAS28 for/at Relapse*	*Events*	*Total Number*	*Event%*
1	Quinn et al., 2005 [32]	–	0.5	–	I	M	10.6	12	–	3	10	30
2	Nawata et al., 2008 [33]	DAS28-ESR < 2.6 after 24 weeks of TNFi therapy	2.4	6.6	I	C; M	6 to 12	14.2	–	5	9	55.55 (If follow-up is 12 months)
3	Brocq et al., 2009 [34]	DAS28 < 2.6 after TNFi therapy for 6 months	11.3	5.54	I, 5%; A, 25%; E, 75%	M, L	40.25	12	> 3.2	15	20	75
4	Tanaka et al., 2010 [35]	DAS28 < 3.2 during > 24 weeks; Prednisolone therapy < 5 mg/day	5.9	5.5	I	M, P	–	12	> 3.2	46	102	45.1
5	van den Broek et al., 2013 [36]	DAS44 < 2.4 for 6 months	1.9	1.3	I	–	11	12	> 2.4*	50	108	48
6	van der Maas et al., 2012 [37]	DAS28 > 2.6 after TNFi therapy for 6 months	12	–	I	M, CS	67	12	> 1.2 of baseline	20	51	39
7	Harigai et al., 2012 [38]	DAS28-CRP < 2.7	10.3	1.6	A	M, CS	45.8	12	DAS28-CRP > 2.7	15	22	68.18
8	Hirata et al., 2013 [39]	DAS28-ESR < 2.6 after 6 months	7.1	–	A	M	–	6	–	21	50	42
9	Smolen et al., 2013 [40]	DAS28 < 3.2 at 36 months of treatment	6.9	–	E	M	36	12	> 3.2	113	197	57.36
10	Iwamoto et al., 2014 [41]	DAS28 < 2.6	8.2	1.9 (MEDIAN)	I	M, CS	–	6	> 3.2	16	40	40 (initial 42; 2 dropouts)
11	Kádár et al., 2014 [42]	Discontinuation for reasons including remission, low disease activity, or infections	15	3.8	Not mentioned specifically	–	20	15	–	5	33	15.16
12	Tanaka et al., 2015 [43]	DAS28-ESR < 2.6 for > 6 months, steroid free	15	3.8	A	M	20	12	DAS28-ESR > 2.6	27	52	51.92
13	Moghadam et al., 2016 [44]	DAS28 < 3.2 during last 6 months; TNFi therapy ≥1 yr.	12	1.98	A, 51%; E, 40%; I, 5%; G, 3%; C, 1%	M, 82%; M + G, 4%; G, 1%; O, 7%; NONE, 6%	–	12	> 3.2 plus an increase of ≥0.6 over baseline	272	531	51.2
14	NCT00808509 (ADMIRE)	–	10.4	1.98	A	M	–	12	–	13	15	87
15	NCT00858780 (DOSERA)	–	–	–	E	M	–	11	–	2	12	16.66 (Etanercept 50 mg)
16	NCT00858780 (DOSERA) b	–	–	–	E	M	–	11	–	1	12	8.33 (Etanercept 25 mg)

*TNFi* tumour necrosis factor inhibitor, *RA* rheumatoid arthritis, *DMARD* conventional synthetic disease-modifying antirheumatic drugs, *DAS28* disease activity score of 28 joints, *CRP* C-reactive protein, *ESR* erythrocyte sedimentation rate, *A* Adalimumab, *E* Etanercept, *I* Infliximab, *G* Golimumab, *C* Certolizumab, *M* Methotrixate, *G* Glucocorticoids, *C* Corticosteroid, *L* Leflunomide, *P* Prednisolone, *O* other; * = DAS 44

The Jadad score was 3 in four out of the five identified RCTs, and 2 in the remaining RCT by Moghadam et al. (Table 3). The different score in the study by Moghadam et al. was due to its open label randomized study design. All non-RCTs were of high quality (Table 4).

**Table 3:** Jadad score for the RCTs

Sl No.	Study Name	Question 1	Question 2	Question 3	Total Score
1	Quinn et al., 2005	1	1	1	**3**
2	Smolen et al., 2013	1	1	1	**3**
3	Moghadam et al., 2016	1	0	1	**2**
4	NCT00858780 (DOSERA)	1	1	1	**3**
5	NCT00858780 (DOSERA) b	1	1	1	**3**

**Table 4:** Methodological quality of the non-RCTs as per CASP checklist

Sl	Study Name	Clearly focused issue	Recruitment acceptable	Exposure measured accurately	Outcome measured accurately	Identified all confounders	Confounders accounted for	Follow-up complete	Follow up long enough
1	Nawata et al., 2008	Yes	Yes	Yes	Yes	Yes	Yes	Yes	Yes
2	Brocq et al., 2009	Yes	Yes	Yes	Yes	Yes	Yes	Yes	Yes
3	Tanaka et al., 2010	Yes	Yes	Yes	Yes	Yes	Yes	Yes	Yes
4	van den Broek et al., 2011	Yes	Yes	Yes	Yes	Yes	Yes	Cannot tell	Yes
5	van den Massk et al., 2012	Yes	Yes	Yes	Yes	Yes	Yes	Yes	Yes
6	Harigai et al., 2012	Yes	Yes	Yes	Yes	Yes	Yes	Yes	Yes
7	Hirata et al., 2013	Yes	Yes	Yes	Yes	Yes	Yes	Yes	Yes
8	Iwamoto et al., 2014	Yes	Yes	Yes	Yes	Yes	Yes	Yes	Yes
9	Kádár et al., 2014	Yes	Yes	Yes	Yes	Yes	Yes	Yes	Yes
10	Tanaka et al., 2015	Yes	Yes	Yes	Yes	Yes	Yes	Yes	Yes
11	NCT00808509 (ADMIRE)	Yes	Yes	Yes	Yes	Yes	Yes	Yes	Yes

**Fig. 2:**
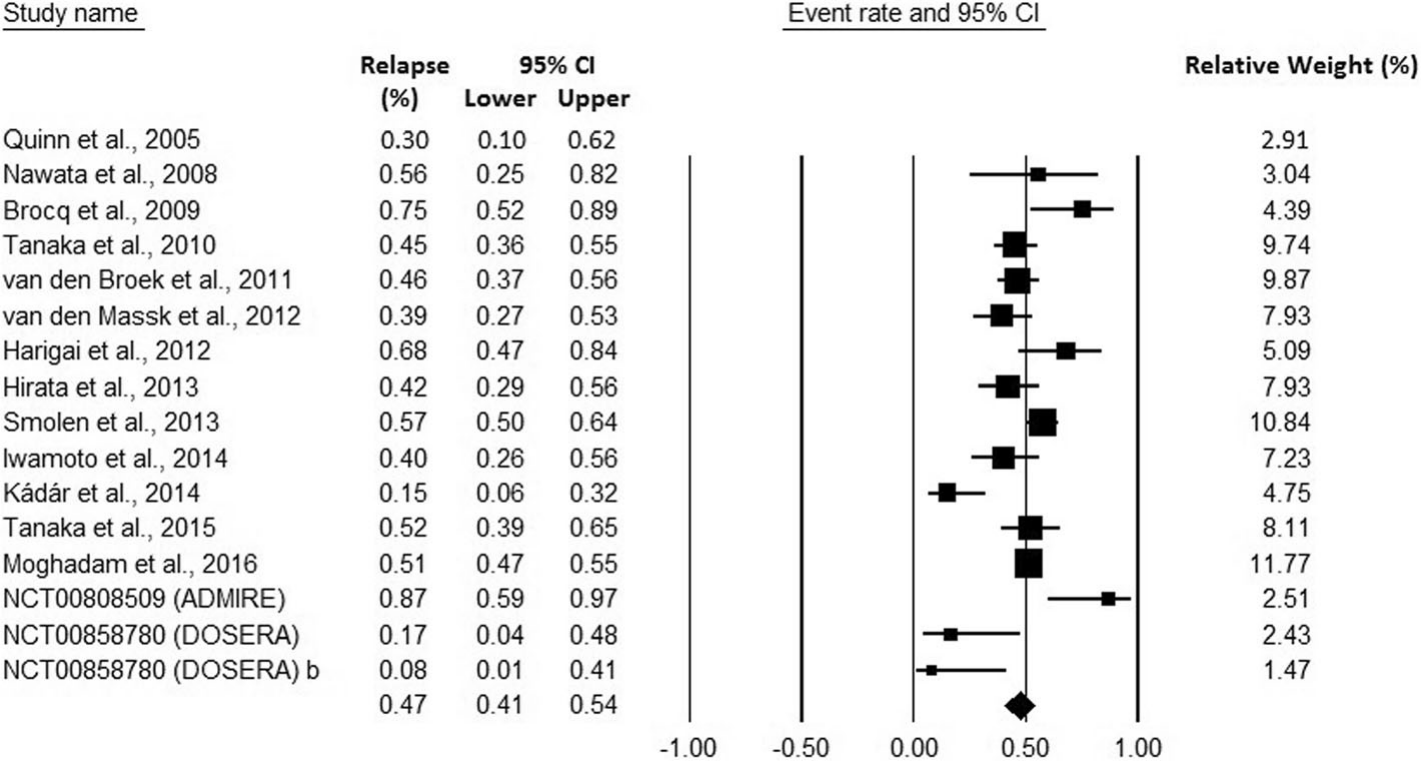
Pooled relapse rates in RA patients after elective withdrawal of anti-TNF therapies

The meta-analysis, conducted in 1264 RA participants from 16 studies, showed that the pooled relapse rate after elective withdrawal of anti-TNF therapy was 0.47; 95% CI 0.41–0.54 (Fig. 2). As significant heterogeneity was observed (Cochrane’s Q-statistics = 48.27; *p*-value: 0.00 and I^2^ = 68.92%), a random-effects-model was used.

**Fig. 3:**
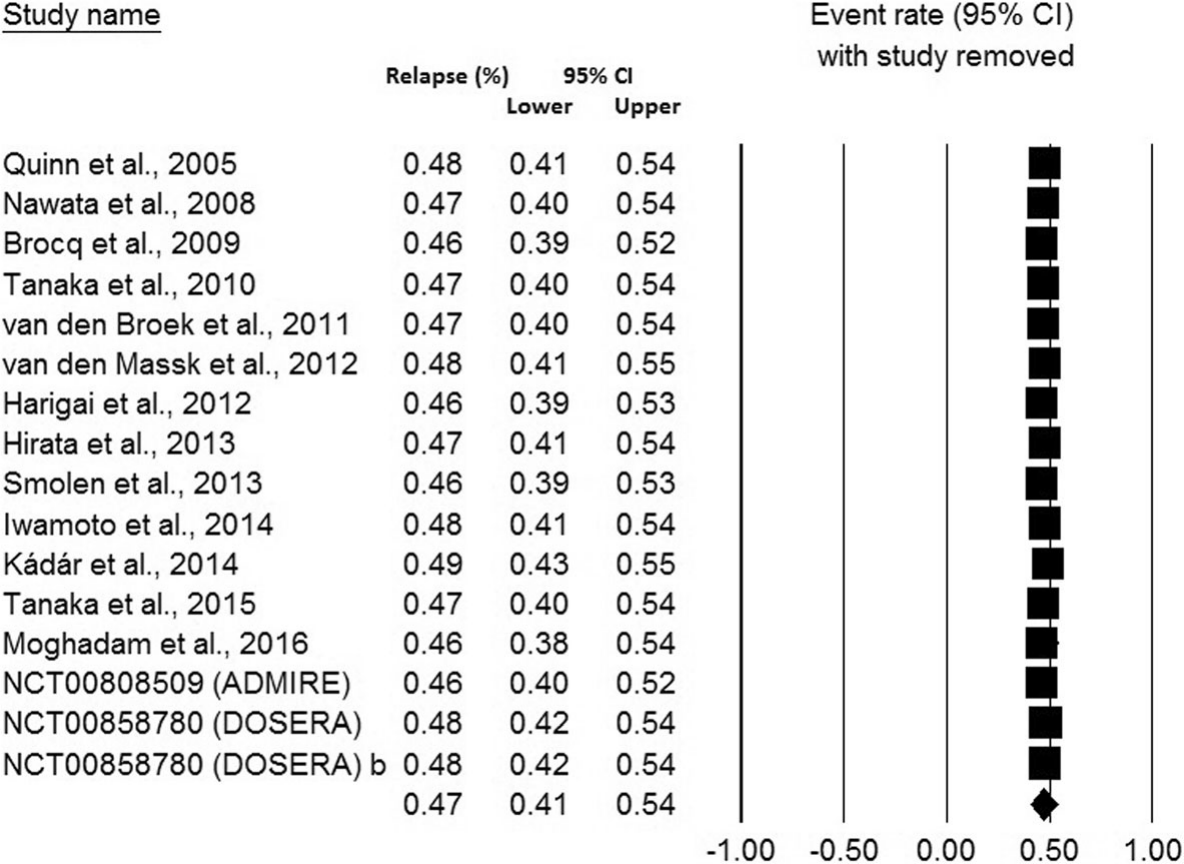
Sensitivity analysis revealing the influence of individual studies on the pooled relapse rates

Sensitivity analysis, assessing the influence of individual studies on the pooled relapse rate by omitting individual studies at each step, suggested that no individual study significantly affected the pooled relapse rate, thus confirming the robustness of the meta-analysis results (Fig. 3).

**Fig. 4:**
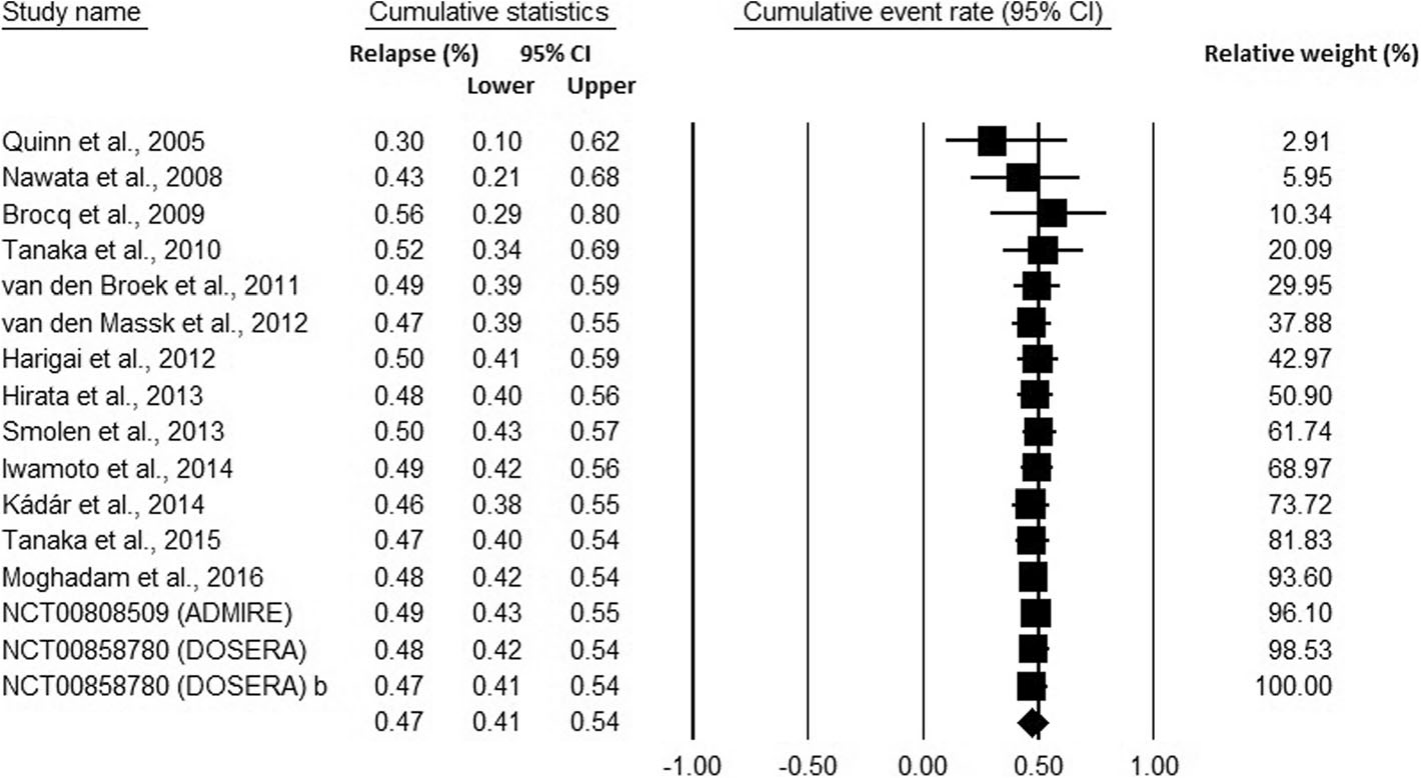
Cumulative meta-analysis of trials studying relapse rates after elective withdrawal of anti-TNF therapy in RA patients

Cumulative meta-analysis of the 16 studies showed that subsequent studies increased the precision of the point estimation (Fig. 4). No substantive change occurred in the direction or magnitude of the estimation.

**Fig. 5:**
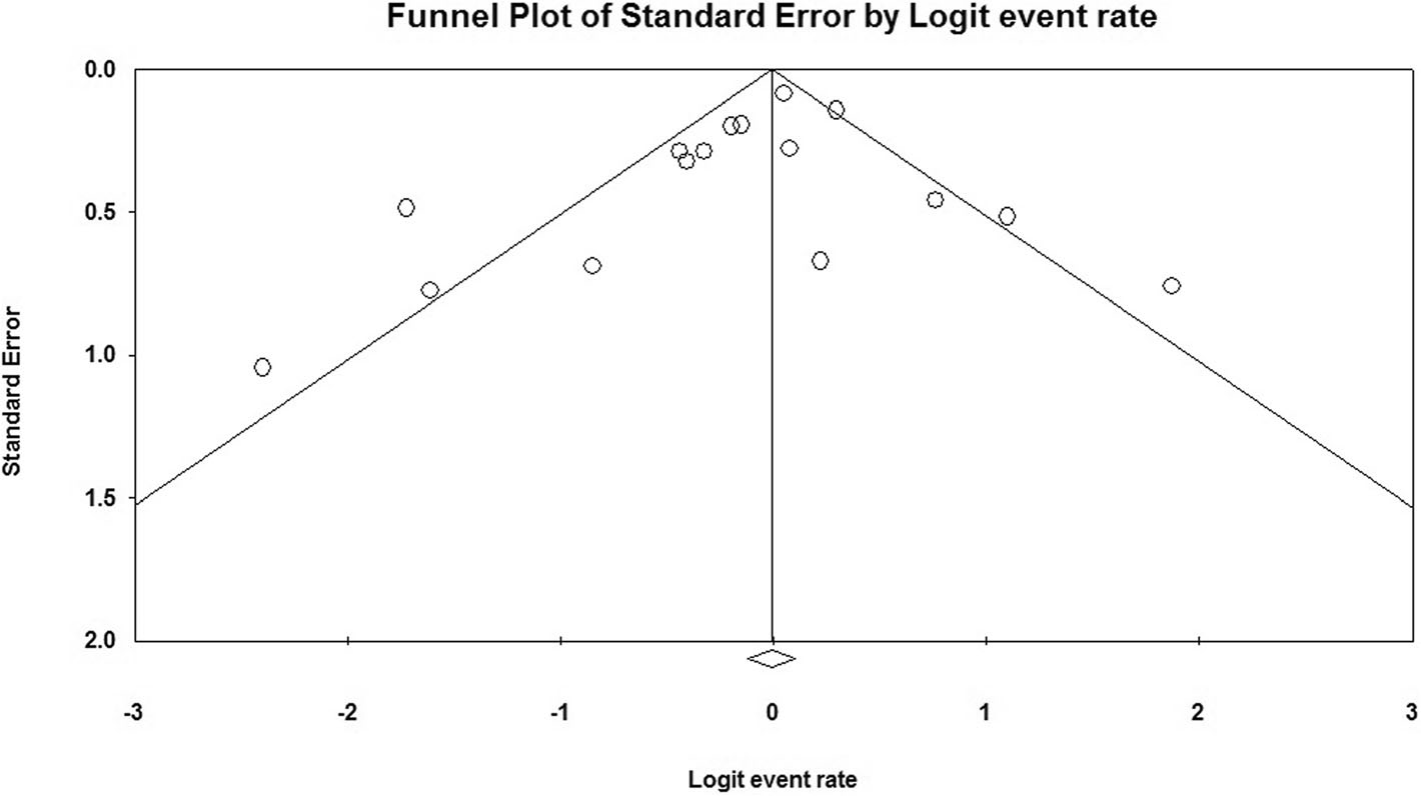
Funnel plot of standard error by log odds ratio

The shape of the funnel plot did not reveal a clear evidence of asymmetry, suggesting no publication bias (Fig. 5). Furthermore, imputation plotted no missing studies on the right side. The trim and fill method, adopted to assess the impact of publication bias, showed that the point estimate and 95% confidence interval for the combined relapse rates remained unchanged after trim and fill test (0.45; 95% CI = 0.38–0.51).

## Discussion

The current pharmacological treatment of RA is based on early intensive therapy with synthetic DMARDs in order to achieve clinical remission. If the latter is not met, further options include increasing the dose of DMARD, adding another synthetic DMARD, or combining the original DMARD with a biologic agent such as TNF-α inhibitors [1]. However, professional guidelines do not provide clear recommendations regarding management strategies once remission, or stable low disease activity, is achieved [1]. Similarly, there is a limited amount of evidence regarding whether, when, how and in whom TNF-α inhibitors can be safely and effectively withdrawn in this context.

In addition to the achievement of remission or stable low disease activity, reasons for TNF-α inhibitor treatment discontinuation include patient preference, cost issues, and adverse effects. Patient surveys have reported that the lack of efficacy and injection reactions, e.g. pain, burning, discomfort, redness, and swelling, were the main factors responsible for treatment discontinuation [20]. However, the uncertainty regarding the magnitude of the overall risk of relapse is an important issue when discussing the option of TNF-α inhibitor treatment withdrawal with RA patients. The availability of robust data regarding the relapse rate in these patients might assist with treatment decisions, as well as informing future guidelines on RA management. This is particularly so as TNF-α inhibitor withdrawal is particularly common in clinical practice. Ramiro et al., investigated the self-reported discontinuation rates of anti-TNF drugs in RA patients (*n* = 2225) in the National Data Bank for Rheumatic Disease, a longitudinal observational study of RA outcomes [21]. Discontinuation of the first TNF-α inhibitor was observed in 1069 (48.0%) of RA patients, with an annual rate of discontinuation of 0.17 (95% CI 0.16 to 0.18). Out of 988 patients who started a second TNF-α inhibitor, 481 (48.7%) reported treatment withdrawal, with an annual discontinuation rate of 0.19 (95% CI 0.17 to 0.21). Older age, smoking, higher comorbidity index, and a higher score of the poly-symptomatic distress scale at baseline independently predicted treatment discontinuation, whereas the concomitant treatment with methotrexate reduced the probability of discontinuation [21]. Similar discontinuation rates have been reported in other observational studies [22, 23].

Our meta-analysis of 16 RCTs on RA patients showed a relatively high (47%) pooled relapse rate after complete elective withdrawal of TNF-α inhibitors. There was significant heterogeneity among studies, however sensitivity analysis ruled out the influence of individual studies on the pooled relapse rate. Furthermore, barring one RCT, all identified studies were considered as having a high methodological quality, i.e. a clearly defined study aim, population, exposure, follow-up and unbiased assessment at the study end-point. In another recent systematic review and meta-analysis by Kuijper et al. the estimated relapse rates were 0.26 (95% CI 0.17 to 0.39) for high-quality studies and 0.49 (95% CI 0.27 to 0.73) for moderate-quality studies. However, unlike our study, focused on complete withdrawal of TNF-α inhibitors, the meta-analysis by Kuijper et al. also included studies reporting dose tapering [24].

A meta-analysis of six trials reported that TNF-α inhibitor treatment continuation, in RA patients in sustained remission or low disease activity, increased the probability of low disease activity (relative risk [RR] = 0.66, 95% CI 0.51–0.84) and remission (0.57, 95% CI 0.44–0.74), and reduced radiographic progression (RR = 0.91, 95% CI 0.85–0.98) [25]. Further, incidence of serious adverse events, serious infection, malignancy, and scores of improvement of tender and swollen joints between these strategies were not significantly different. About half of the patients withdrawing biologicals maintained low disease activity [25]. Similarly, an earlier study in 91 RA patients receiving the TNF-α inhibitor etanercept showed that a significantly higher number of patients stayed in remission with continued therapy vs. treatment withdrawal (52% vs. 13%; *P* = 0.007). The latter group also had a very short time to failure (median of 6 weeks) as compared to those on full dose (48 weeks; *P* = 0.001). Patients on continued therapy were more efficient in regaining remission after a flare-up [9].

The observed pooled relapse rate post-TNF-α inhibitor withdrawal in our study provides important quantitative data that complement existing information regarding relapse rates after discontinuation of other DMARD therapies in RA patients. The latter have been reported to be 40.0–78.9% with penicillamine [26, 27], 66.7% with azathioprine [26, 28], 100% with methotrexate [26, 29], 33.3% with gold [26], and 47.1% with sulphasalazine [26]. However, a direct comparison of relapse rates with various DMARDs is rendered difficult because of the different baseline clinical characteristics, concomitant RA treatment, methods to assess relapse, and follow-up in individual studies. Furthermore, the RA treatment strategies investigated in these relatively old studies are quite different from those recommended by current professional guidelines.

All the studies included in this analysis, employing rigid criteria for measuring disease activity and monitoring remission, used improvement in DAS28 scoring system. This is a widely used and recommended criterion [30]. However, this in itself could be one of the key reasons for higher relapse rates. A recent conference paper showed that total dependence on DAS28 for monitoring complete remission may not be a reliable method to ensure that patients remain in remission [31]. This study used DAS28 to monitor disease activity and ACR/EULAR 2010 criteria to measure response to therapy coupled with MRI for dominant joint erosions. 73% of the patients showing improvement in DAS28 score after 12 months also showed decrease in erosions, while 24% had increased erosions. 41% patients who attained remission as per EULAR score also had increased erosions. 40% of all the patients, despite showing improvements in DAS28 score, continued to undergo progressive erosive arthritis. It is highly likely that patients from the studies included in our analysis also had undergone continued erosions even after DAS28 improvements. As a result, it is possible that the relapse rate in ‘real-life’ is even higher than that (47%) reported in our meta-analysis.

The results of our meta-analysis provide much needed information regarding the magnitude of the overall risk of relapse in RA patients receiving TNF-α inhibitor therapy, where treatment withdrawal is being considered by the patient and/or the treating physician. However, some caution is required when translating these results into routine clinical practice because of the differences between studies in the treatment duration with TNF-α inhibitors at baseline, the criteria used to define TNF-α inhibitor withdrawal eligibility and relapse, the concomitant treatment with other DMARDs, and the duration of follow-up. Further limitations include the relatively small sample size of the selected studies and the fact that relapse rates, not their severity, were investigated.

## Conclusions

Our study shows that elective TNF-α inhibitor withdrawal in RA patients is associated with a relatively high relapse rate. This information should be taken into account when considering this management strategy. Further studies are required to identify whether specific patient characteristics, TNF-α inhibitors discontinued, or concomitant DMARDs independently predict the risk of relapse in this patient group.

## Abbreviations

ACR: American College of Rheumatology
ANA: Antinuclear antibody
CASP: The Critical Appraisal Skills Programme
CI: Confidence Interval
DAS: Disease Activity Score
DMARD: Disease-modifying antirheumatic drug
EULAR: European League Against Rheumatism
MRI: Magnetic Resonance Imaging
NSAID: Nonsteroidal anti-inflammatory drug
RA: Rheumatoid arthritis
RCT: Randomised controlled trials
TNF-α: Tumor necrosis factor alpha
US: United States
WHO: World Health Organisation
